# Preclinical evaluation of the PARP inhibitor BMN-673 for the treatment of ovarian clear cell cancer

**DOI:** 10.18632/oncotarget.14011

**Published:** 2016-12-17

**Authors:** Paul M Wilkerson, Konstantin J Dedes, Eleftherios Pierre Samartzis, Ioannis Dedes, Maryou B Lambros, Rachael Natrajan, Arnaud Gauthier, Salvatore Piscuoglio, Chantal Töpfer, Vesna Vukovic, Frances Daley, Britta Weigelt, Jorge S Reis-Filho

**Affiliations:** ^1^ The Breakthrough Breast Cancer Research Centre, Institute of Cancer Research, London, SW3 6JB, UK; ^2^ Department of Gynaecology, University Hospital of Zurich, 8091 Zurich, CH; ^3^ Department of Pathology, Memorial Sloan Kettering Cancer Center, New York, NY 10065, USA

**Keywords:** ovarian clear cell carcinoma, homologous recombination, PTEN, PARP inhibitors

## Abstract

**Purpose:**

To determine if models of ovarian clear cell carcinomas (OCCCs) harbouring defects in homologous recombination (HR) DNA repair of double strand breaks (DSBs) are sensitive to cisplatin and/or PARP inhibition.

**Experimental Design:**

The HR status of 12 OCCC cell lines was determined using RAD51/γH2AX foci formation assays. Sensitivity to cisplatin and the PARP inhibitor BMN-673 was correlated with HR status. BRCA1, BRCA2, MRE11 and PTEN loss of expression was investigated as a potential determinant of BMN-673 sensitivity. A tissue microarray containing 50 consecutive primary OCCC was assessed for PTEN expression using immunohistochemistry.

**Results:**

A subset of OCCC cells displayed reduced RAD51 foci formation in the presence of DNA DSBs, suggestive of HR defects. HR-defective OCCC cells, with the exception of KOC-7c, had higher sensitivity to cisplatin/ BMN-673 than HR-competent OCCC cell lines (Log10 SF50 –9.4 (SD +/− 0.29) vs –8.1 (SD +/− 0.35), mean difference 1.3, *p* < 0.01). Of the cell lines studied, two, TOV-21G and KOC-7c, showed loss of PTEN expression. In primary OCCCs, loss of PTEN expression was observed in 10% (5/49) of cases.

**Conclusions:**

A subset of OCCC cells are sensitive to PARP inhibition *in vitro*, which can be predicted by HR defects as defined by γH2AX/RAD51 foci formation. These results provide a rationale for the testing of HR deficiency and PARP inhibitors as a targeted therapy in a subset of OCCCs.

## INTRODUCTION

Ovarian clear cell carcinoma (OCCC) is an aggressive histological subtype of epithelial ovarian cancer (EOC) with a higher rate of *de novo* resistance to platinum-based chemotherapy than high-grade serous EOCs [1, 2]. OCCCs have been shown to constitute a distinct subtype of EOCs histologically and genetically. Unlike high-grade serous carcinomas, OCCCs usually lack *TP53* mutations [3] and germline or somatic *BRCA1* or *BRCA2* mutations [4]. In contrast, OCCCs are characterised by the presence of *ARID1A* mutations [5], activating *PIK3CA* mutations [6], loss of PTEN expression [6, 7], and amplification of *PPM1D* [8]. It should be noted, however, that there is evidence to suggest that OCCCs constitute a heterogeneous group of cancers at the genetic level, exemplified by the existence of subgroups harbouring distinct constellations of gene copy number alterations [9] and a varied repertoire of somatic mutations [5].

Despite the progress in the understanding of the molecular basis of OCCCs, patients with this disease are still managed with chemotherapy based on platinum salts and taxanes. Given the reported aggressive clinical behaviour and generally poor response rates to these conventional chemotherapy regimens, patients with OCCCs have been shown to have a worse outcome than those with other types of EOCs [9, 10]. It should be noted, however, that the heterogeneity of OCCCs is also apparent in terms of its response to specific therapeutic agents, as there are models of OCCCs that have been shown to be sensitive to platinum salts [2].

Poly(ADP) ribose polymerase (PARP) inhibitors constitute a new class of targeted therapeutic agents that have shown great promise in preclinical studies and phase I/ II clinical trials based on the principle of synthetic lethality [11, 12], PARP inhibitors selectively target cells that lack competent homologous recombination (HR) DNA repair in the presence of DNA double-strand breaks (DSBs) [13]. PARP inhibition leads to an impairment of the ability of cells to repair DNA single-strand breaks by base excision DNA repair. In cells treated with PARP inhibitors, single-strand breaks are not repaired and during S-phase, these lead to replication fork stalling and collapse, ultimately resulting in DNA DSBs [13]. In normal cells, these DSBs are repaired by HR DNA repair. In cancers with dysfunctional HR, such as those lacking BRCA1 [14], BRCA2 [14], PTEN [15] and RAD51D [16] function, PARP inhibitor-induced DSBs cannot be corrected by HR, and are repaired by error prone mechanisms (e.g. non-homologous end-joining), which result in high levels of genetic instability and eventually cell death. Defects in HR can be associated with inactivation of multiple components of the HR pathway [17, 18]. In particular, BRCA1 and BRCA2 loss of function have been shown to lead to dysfunctional HR in cancer cells [15, 18] whereas the association between PTEN loss of function and dysfunctional HR is more controversial [19]. MRE11 loss of expression has recently been shown to be associated with increased PARP inhibitor sensitivity in prostate, colorectal and endometrial cancer cells [20, 21].

Consistent with the results from preclinical models for the PARP-inhibitor BMN-673 [22, 23], clinical trials testing the efficacy of PARP inhibitors, have yielded promising results in patients with advanced *BRCA1* or *BRCA2* hereditary breast and ovarian cancers [24–31], but also in sporadic high-grade serous EOC [32, 33], which often harbour somatic inactivation of genes involved in HR DNA repair [34]. Pre-clinical studies [15] and clinical evidence from the analysis of a patient with advanced endometrial cancer [35] suggests that tumours with loss of PTEN expression, a recurrent aberration in OCCC [6, 7], may be sensitive to PARP inhibitors.

Given that OCCCs are heterogeneous at the molecular level and that a subgroup of these cancers are sensitive to platinum-based chemotherapy, we hypothesised that a subset of OCCCs may have dysfunctional HR DNA repair of DNA DSBs and that this defect would result in sensitivity to platinum salts and PARP inhibitors. To address these hypotheses, we analysed a panel of 12 OCCC cell lines and a series of 50 primary OCCCs i) to define the prevalence of dysfunctional HR DNA repair, ii) to determine if subgroups of OCCCs are sensitive to platinum salts and/or the PARP inhibitor BMN-673, iii) to determine if HR DNA repair defects would be associated with loss of PTEN function, and iv) to determine the prevalence of PTEN loss of expression in a cohort of primary OCCCs.

## RESULTS

### A subset of OCCC cells is unable to elicit RAD51 foci in the presence of DNA DSBs

**Figure 1:**
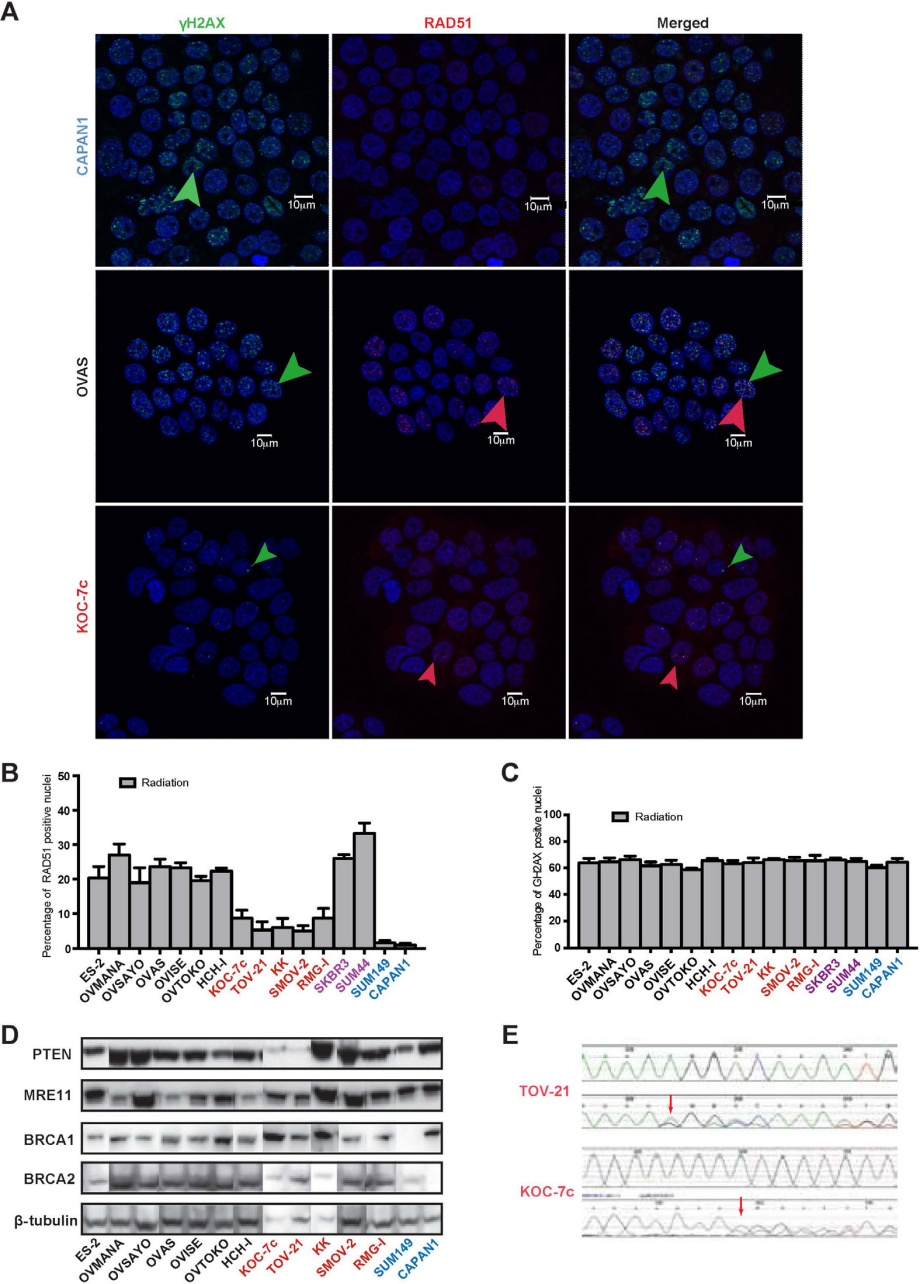
A subset of OCCC cells is unable to elicit RAD51 foci in the presence of DNA DSBs A panel of 12 OCCC cell lines were subjected to RAD51 foci formation assays to determine their homologous recombination (HR) function in the presence of DNA double strand breaks (DSBs). Cell lines were treated with ionising radiation before immunofluorescent detection of γH2AX foci (markers of DNA DSBs) and RAD51 foci (markers of competent HR) was undertaken (**A**). Akin to CAPAN1 (*BRCA2*-mutant) and SUM149 (*BRCA1*-mutant) control cells, five OCCC cell lines showed reduced RAD51 foci formation (i.e. TOV-21G, KOC-7c, SMOV-2, RMG-1, and KK) (**B**). The remaining seven OCCC cell lines displayed RAD51 foci formation at similar levels to HR-competent SKBR3 and SUM44 cells (B). All cell lines showed similar levels of γH2AX foci formation (**C**). Western blot analysis of PTEN, MRE11, BRCA1 and BRCA2 in 12 OCCC cell lines. Beta-tubulin was employed as loading control. Two cell lines (TOV-21G and KOC-7c) showed loss of PTEN expression (**D**). PTEN Sanger sequencing traces of TOV-21G (top) and KOC-7c (bottom), which both harboured a *PTEN* frameshift mutation (c.795het_delA in TOV-21G and c.968het_delA in KOC-7c), red arrows, (**E**). The top sequencing trace in each panel represents the wild type sequence, while the cell line sequence showing the mutation is shown below it. In all panels, cell lines with reduced RAD51 foci formation (HR defective) are marked in red, those with normal RAD51 foci formation (HR competent) are marked in black, those with BRCA1 or BRCA2 loss of function are marked in blue, and those previously documented to be HR competent in purple.

We sought to determine if OCCC cells would harbour defects in HR. The inability of cancer cells to elicit RAD51 foci in the presence of DNA DSBs was used as a surrogate for dysfunctional HR as previously described [14, 15, 41]. We first assessed the ability of OCCC cells to elicit RAD51 foci formation in the presence of ionising radiation-induced DNA DSBs (Figure 1A–1C). As expected, SUM149 and CAPAN1 cells were unable to elicit RAD51 foci following exposure to DNA damaging agents, given that they harbour deleterious *BRCA1* and *BRCA2* mutations, respectively (Figure 1A and 1B) [14, 15, 41]. Although none of the OCCC cells completely lacked the ability to elicit RAD51 foci, KOC-7c, TOV-21G, KK, RMG-1, and SMOV-2 cells had significantly lower proportions of RAD51-positive nuclei in response to ionising radiation than HR competent cancer cells (i.e. SKBR3 and SUM44, Mann-Whitney *U* Test, *p <* 0.01, Figure 1A and 1B). These findings suggest that a subset of OCCC cells may lack competent HR DNA repair of DSBs.

### A subset of OCCC cells unable to elicit RAD51 foci in the presence of DSBs harbours *PTEN* gene mutations

**Figure 2:**
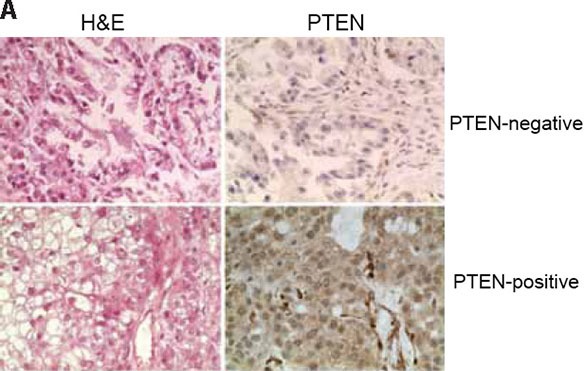
PTEN expression in primary OCCCs Using a validated antibody to PTEN, and previously reported scoring systems, PTEN expression was assessed in a panel of 50 primary OCCCs in a previously constructed tissue microarray. Cases were reviewed by at least two pathologists and discordant scores reassessed on whole tissue sections. Complete loss of PTEN expression was identified in 5/49 analysable cases (10%). Representative haematoxylin & eosin micrographs (left) and PTEN immunohistochemistry (right) of a PTEN-negative (top) and a PTEN-positive OCCC.

As BRCA1, BRCA2, PTEN and MRE11 loss has been associated with dysfunctional HR and/or PARP inhibitor sensitivity, we assessed the expression of BRCA1, BRCA2, PTEN and MRE11 in 12 OCCC cell lines included in this study by western blotting. All OCCC cells expressed similar levels of BRCA1, BRCA2 and MRE11 protein (Figure 1D). In addition, all OCCC cells with competent HR as defined by the RAD51 foci formation assay expressed PTEN protein. Out of the five cell lines with reduced RAD51 foci in the presence of DSBs, however, two, TOV-21G and KOC-7c, showed loss of PTEN expression (Figure 1D) and harboured *PTEN* frameshift mutations (TOV-21G, c.795_795delA; KOC-7c, c.968_968delA; Figure 1E). None of the cell lines tested displayed *PTEN* homozygous deletions and both PTEN-null cell lines (TOV-21G and KOC-7c) harboured two copies of the *PTEN* locus as defined by FISH (data not shown). No *BRCA1* was detected in these cell lines (Supplementary Table S3). Taken together, these data suggest that a subset of OCCC cell lines harbour defects in HR, some of which are associated with loss of PTEN expression. Consistent with these data, when a cohort of 50 primary OCCC tumour samples were assessed for PTEN loss of expression by immunohistochemistry (Figure 2), 5/49 analysable cases were found to harbour PTEN loss (10%, Supplementary Table S1). This is less frequent than previously reported for OCCCs (27.5–37.5%) [7, 43].

### Sensitivity of OCCC cell lines to cisplatin and BMN-673 is associated with HR functional status

**Figure 3:**
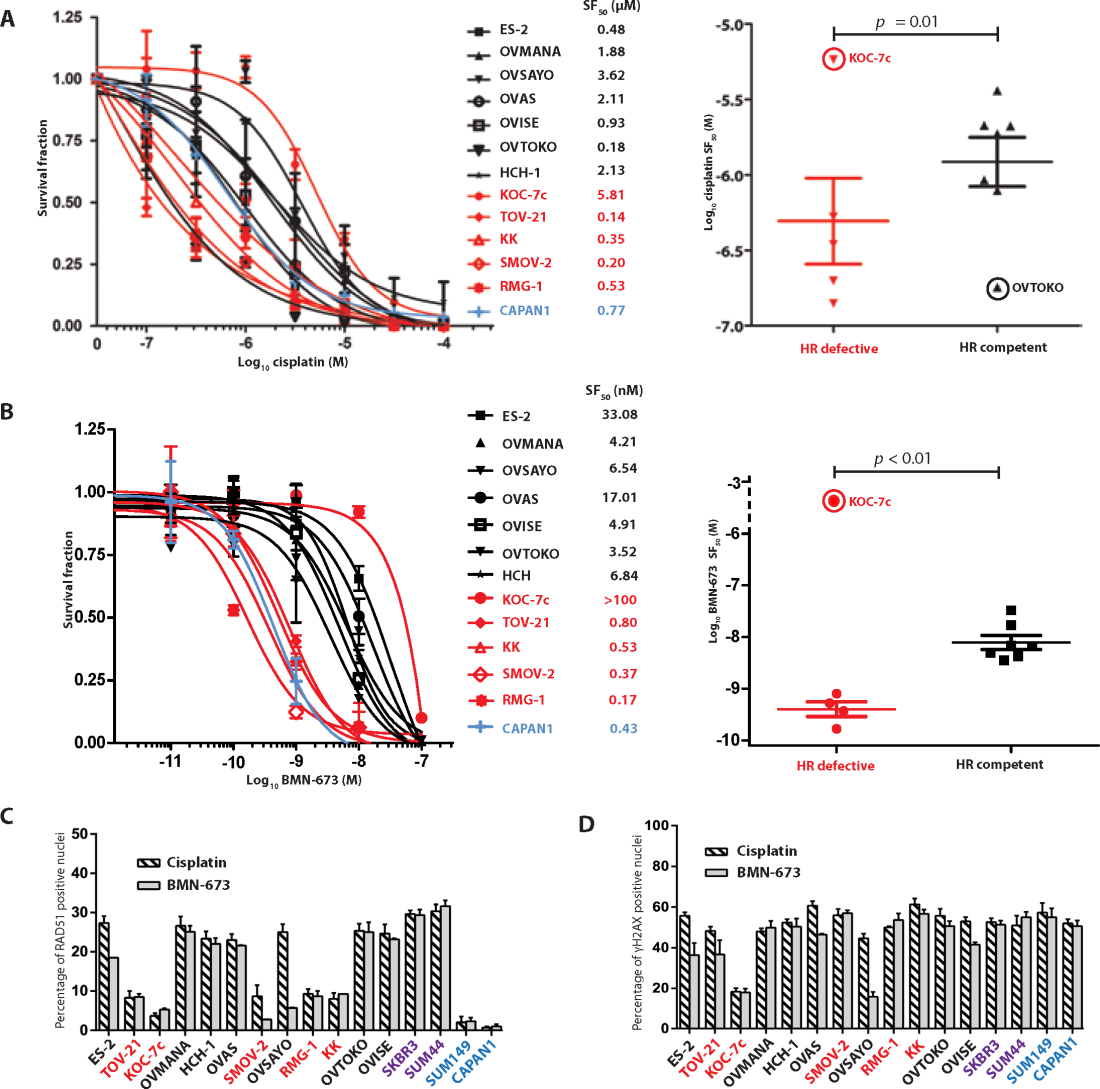
Sensitivity of OCCC cell lines to cisplatin and BMN-673 can be predicted by HR functional status and PTEN status Given the recognised sensitivity of cells with defects in HR to agents that cause DNA double strand breaks, the panel of 12 OCCC cell lines were subjected to treatment with cisplatin or the PARP inhibitor BMN-673. With the exception of KOC-7c, all HR defective cell lines showed dose-response curves to cisplatin (**A**) and BMN-673 (**B**) with sensitive profiles, while all HR competent cell lines (with the exception of OVTOKO) showed dose-response curves with resistant profiles. CAPAN1 cells were used as controls for this experiment. When outliers were excluded (KOC-7c, circled in scatter plots, A and B as well as OVSAYO circled in scatter plot B), HR defective cell lines had cisplatin and BMN-673 SF_50_ concentrations that were significantly lower than those of HR competent cell lines (Mann-Whitney *U* test, *p <* 0.01) Assessment of RAD51 (**C**) and γH2AX (**D**) foci following treatment with cisplatin and BMN-673 demonstrated that HR defective cell lines had a lower rate of RAD51 foci formation compared to HR competent cells (C). KOC-7c, did not form γH2AX to the same degree as other treated cells (D), suggesting cisplatin and BMN-673 may be subject to drug efflux. In all panels, HR defective cell lines are marked in red, HR competent cell lines are marked in black, those with *BRCA1/2* mutations are marked in blue, and those previously documented to be HR competent in purple. Scatter plots in A and B are annotated with the mean and standard error of mean (SEM).

Given that a subset of OCCC cells was unable to elicit RAD51 foci in the presence of DNA DSBs, we sought to define whether these cell lines would show a higher sensitivity to cisplatin and PARP inhibitors than the remaining OCCCs. Interestingly, cell lines with low levels of RAD51 foci formation upon treatment with ionising radiation were sensitive to cisplatin and BMN-673 (Figure 3A and 3B), with survival fractions 50 (SF_50_s) similar to those of the *BRCA2*-mutant CAPAN1 cells, with the exception of KOC-7c. On the other hand, OCCC cells able to elicit RAD51 foci upon treatment with ionising radiation were relatively resistant to cisplatin and BMN-673 (Figure 3A and 3B).

Given that KOC-7c cells elicited relatively low numbers of RAD51 foci upon treatment with ionising radiation, but displayed a relative resistance to cisplatin and BMN-673 (Figure 3A and 3B), we sought to define the ability of these cells to elicit RAD51 and γH2AX foci upon treatment with these agents. Upon treatment with cisplatin and BMN-673, KOC-7c, TOV-21G, KK, RMG-1, and SMOV-2 cells displayed significantly lower proportions of RAD51-positive nuclei than the remaining OCCC cells (7.1 (SD +/− 1.37) vs 18.8 (SD +/− 3.31), *p <* 0.05) (Figure 3C). Importantly, however, the assessment of γH2AX foci formation upon treatment with cisplatin or BMN-673 in these cells revealed a lower proportion of γH2AX-positive nuclei in KOC-7c cells than in the remaining OCCC cell lines (17.2 vs 43.9 (SD +/− 7.68), *p <* 0.05) (Figure 3D). These observations provide strong circumstantial evidence that cisplatin and BMN-673 are not causing DNA damage in KOC-7c cells.

## DISCUSSION

OCCCs have been shown to constitute a heterogeneous group of tumours in terms of their genetic features [9] and response to therapy [44]. Here we demonstrate that OCCCs are also heterogeneous in regards to their ability to elicit competent HR DNA repair. A subset of OCCC cells exhibited impaired HR DNA repair of DSBs, as determined by RAD51 foci formation assays, and were shown to be sensitive to cisplatin and BMN-673. In addition, our results also provided evidence to suggest that PTEN loss of function may be associated with impaired HR DNA repair in a subset of OCCC cells and was found to be lost in 10% of primary OCCCs.

The role of PTEN loss of function in HR DNA repair of DSBs is controversial. Studies have failed to demonstrated a significant association between PTEN loss of function [45, 46] and either sensitivity to PARP inhibitors or HR defects have not yet provided a definite mechanism for this association [15, 47]. Possible mechanisms include the role of PTEN as a modulator of RAD51 transcription [48]. Importantly, the impact of PTEN loss of function in HR DNA repair of DSBs may be context dependent. While in endometrial cancer [15, 35] and gliomas [47] PTEN loss of function results in impaired HR DNA repair and sensitivity to PARP inhibitors, similar effects have not been observed in non-small cell lung cancer and prostate cancer [20, 49]. Endocrine factors, in particular estrogen levels may also play a role, in sensitizing PTEN-null endometrial cancer cells to PARP inhibitors [50]. It is also plausible that the chronology of the loss of PTEN function may determine its impact on HR DNA repair. In endometrial cancers, loss of PTEN is an early event, whereas in prostate cancer, there is evidence to suggest that PTEN loss is a late event [19]. More recent data in prostate cancer suggest that the effects of PTEN loss on cell growth and response to PARP inhibition are context dependent [51]; in a series of *in vitro* and *in vivo* experiments using *pten* heterozygous, *pten* null, with or without *tp53* loss, mouse embryonic fibroblasts (MEFs) or mice, it was demonstrated that in a p53 proficient setting, treatment of PTEN–deficient cells with PARP inhibitors leads to a senescence response. By comparison, upon loss of p53, the response is more apoptotic. Furthermore, the group demonstrated that treatment with PARP inhibitor leads to AKT activation through the PI3K cascade, which can be mitigated with combination therapy with dual PI3K inhibitors and PARP inhibitors.

Our results are consistent with those of previous reports suggesting that PTEN loss of function is associated with defective HR-mediated repair of DNA DSBs, as defined by RAD51 foci formation [15, 18]. Both OCCC cell lines studied here that harbour PTEN loss of function (TOV-21G and KOC-7c) exhibit impaired HR DNA repair as determined by RAD51 foci formation. In the light of the recent data in prostate cancer discussed above, it is possible that these two events (PTEN loss of function and impaired HR DNA repair) may not be causally linked in isolation; PTEN loss of function may be a bystander event in these HR defective OCCC cell lines, or a hitherto as yet undiscovered epistatic interaction may be required for PTEN loss of function to be mechanistically linked to impaired HR DNA repair. Further functional studies assessing PTEN loss of function in OCCC are required to clarify this issue. Given that aberrations in several other genes in the HR pathway can lead to defects in HR [18], it is plausible that other HR DNA repair-related genes may be lost in a subgroup of OCCCs [24, 25, 52]. Through a reanalysis of the results by Tan et al. [9], we have ruled out *EMSY* amplification [41] as a potential cause of HR deficiency in these cells, as none of them harboured this genetic aberration (data not shown). Further studies to define the basis of the deficient HR DNA repair in these cells are warranted.

Contrary to the observations derived from the experiments performed with BMN-673, where all BMN-673-sensitive cell lines were HR DNA deficient, OVTOKO cells exhibited an exquisite sensitivity to cisplatin, despite being HR competent. The fact that OVTOKO was resistant to BMN-673 suggests this cisplatin sensitivity is not mediated by a defect in HR, and that impaired HR-mediated repair of DNA DSBs is not the only determinant of cisplatin sensitivity [53, 54].

This study has several limitations. Although the OCCC models tested in this study have been shown to recapitulate the genetic and transcriptomic features of primary OCCCs [8, 9], seven of the 12 cell lines assessed in this study had cisplatin SF_50_s comparable to those CAPAN1 (sub-micromolar concentrations). Hence, there might be an enrichment for chemotherapy–sensitive OCCCs in this panel of cell lines.

In conclusion, we have identified a subset of OCCC cell lines that harbour defects in HR-mediated repair of DNA DSBs. This preclinical study provides a rationale for the assessment of HR deficiency *in vivo* to potentially identify a role of PARP inhibitors in this histological subset.

## MATERIALS AND METHODS

Further details of all the methods described here are found in the Supplementary Methods.

### RAD51 and γH2AX foci assessment

To determine if cancer cells would have dysfunctional HR repair of DNA DSBs, Nuclear γ-H2AX and RAD51 foci formation was employed as previously described [15]. Details of the methods used are described in the Supplementary Methods. To determine HR status, RAD51 foci formation in response to 10 Gy of ionizing radiation was assessed. The differential response of HR competent and HR defective cells to either 10 μM cisplatin for 6 hrs or 10 μM BMN-673 for 12 hrs was then determined.

### Immunoblotting

Western blotting was performed as previously described [36], using anti-β-tubulin (ab6046, Abcam, Cambridge, UK), anti-PTEN (138G6, Cell Signaling Technology, Danvers, MA, USA), anti-BRCA2 (Ab-1 [OP95], Calbiochem/Merck, Nottingham, UK) and anti-BRCA1 (C-20, Santa Cruz Biotechnology) antibodies.

### Sanger sequencing

Sequencing of the full-length cDNA of PTEN was performed for all cell lines as previously described [8]. Mutations were confirmed by a repeat PCR from a new cDNA from the cell line harbouring the mutation and by sequencing of forward and reverse strands.

### Assessment of *PTEN* copy number in OCCC cells

To determine *PTEN* copy number in OCCC cells and if any OCCC cells harboured homozygous deletions of *PTEN*, fluorescence *in situ* hybridisation (FISH) was performed as previously described [9, 15]. The Vysis *PTEN/CEP10* dual colour FISH probe (Abbott Molecular, Des Plaines, IL, USA) was hybridised to slides as previously described [37].

### Cisplatin sensitivity assays

Cisplatin sensitivity assays were carried out in 96 well plates, in triplicate, as previously described [15]. OCCC cells were grown for at least seven days, using CAPAN1 as a HR DNA repair-deficient and cisplatin- sensitive control [38–40]. Cells were treated with DMSO (control) or cisplatin (serial dilutions; 10^–7^M to 10^–4^M) (Sigma Aldrich). CellTiter Glo Luminescent Cell Viability Assay (Promega, UK) was used according to manufacturer's instructions to determine the survival fraction of cisplatin vs DMSO-treated cells.

### BMN-673 sensitivity assays

BMN-673 sensitivity was determined using colony formation assays (CFAs) in six well plates in triplicate, as previously described [15]. Cells were treated with 10^–7^M to 10^–11^M BMN-673 every 2 days. After 14 days cells were fixed and cell number quantified with a sulphorhodamine B assay, as previously described [15, 41].

### Patient samples and tumour characteristics

Formalin-fixed paraffin-embedded (FFPE) samples from 50 consecutive primary OCCCs were retrieved from the pathology files of The Royal Marsden Hospital, London, The Edinburgh Royal Infirmary, The Royal Hospital Group, Belfast and The Hammersmith Hospital, London, as previously described [8]. Patient demographics and tumour characteristics are summarised in Supplementary Table S1, in line with REMARK guidelines (Supplementary Table S2). Samples were anonymised prior to analysis and the study approved by local ethical committees of the authors’ institutions. All cases were reviewed and diagnosis of clear cell carcinoma confirmed as previously described [8]. Tissue microarrays (TMAs) were constructed from paraffin blocks with triplicate 0.6 mm tumour cores, containing 50 samples and normal tissue controls, as previously described [9]. Surgical and adjuvant therapy are described in Supplementary Table S1.

### Immunohistochemistry (IHC)

Representative 3 μm-thick sections of the TMA described above were subjected to immunohistochemistry (IHC) using an antibody against PTEN (6H2.1 Dilution 1:100, Antigen retrieval, Dako target retrieval solution, pH9, 20 min 97C° DAKO, Glostrup, Denmark). The protocol for PTEN immunohistochemical analysis was previously validated [15]. Immunohistochemical analysis was interpreted by at least two pathologists (AC-F, AG and/ or JRF) using a semi-quantitative scoring system. Nuclear and cytoplasmic PTEN expression was scored using the Quick Score method [42], with cases defined as PTEN negative if nuclear expression was absent (i.e. Quick Score = 0). Cases with discordant scores between replicate cores, or those with missing cores from the TMA were assessed on full sections by the three pathologists on a multi-headed microscope and a consensus score for each was rendered.

### Statistical analysis

All statistical analyses were carried out in Prism 6.05 (Graphpad Software Inc., California, USA). Details of statistical analyses are described in the Supplementary Methods. A two-tailed *p value* < 0.05 was considered significant.

## SUPPLEMENTARY MATERIALS AND TABLES


