# Supplementary material for: Preclinical evaluation of the PARP inhibitor BMN-673 for the treatment of ovarian clear cell cancer
Source: Oncotarget. 2016 Dec 17;8(4):6057–66. doi: 10.18632/oncotarget.14011 (PMC5351612; doi:10.18632/oncotarget.14011)
Supplement: Supplementary file 1 [file oncotarget-08-6057-s001.pdf]

Preclinical evaluation of the PARP inhibitor BMN-673 for the treatment of ovarian clear cell cancer

Supplementary Materials

SUPPLEMENTARY METHODS

Cell lines and growth conditions

TOV-21G and ES2 were obtained from the American Type Culture Collection. SMOV-2, RMG-1, KOC-7C, HCH-1, OVAS, OVISE, OVTOKO, OVMANA, OVSAYO and KK were courtesy of Dr. Hiroaki Itamochi (Tottori University School of Medicine, Yonago, Japan). All cell lines were grown as previously described [1]. All cell lines were shown to be mycoplasma-free and authenticated by means of Short Tandem Repeat (STR) analysis (PowerPlex® 1.2 System, Promega, WI, US) as previously described [2, 3]. STR profiles were matched to the German Collection of Microorganisms and Cell Cultures (DSMZ)–database (www.dsmz.com).

RAD51 and γH2AX foci assessment

To determine if cancer cells would have dysfunctional HR repair of DNA DSBs, Nuclear γ-H2AX and RAD51 foci formation was employed as previously described [4]. Cells were grown onto L-lysine–coated coverslips and exposed to either 10 µM cisplatin for 6 hrs or with 10 µM BMN-673 for 12 hrs, respectivley 10 Gy of ionizing radiation. After 6 hours, cells were fixed, permeabilized, and co-immunostained with primary antibodies targeting RAD51 (H-92, Santa Cruz Biotechnology, Santa Cruz, CA, USA) and γ-H2AX ((#05-636, Millipore, Billerica, MA, USA)) or Ki67 (MIB-1, Dako, Cambridgeshire, UK). Nuclei were counterstained with 4′,6-diamidino-2-phenylindole (DAPI). The presence of γ-H2AX and RAD51 foci was evaluated in a minimum of 100 cells in three independent experiments. The HR-deficient CAPAN1 (647delT *BRCA2*-mutant) and SUM149 (2288delT *BRCA1*-mutant) cells lines were employed as negative controls [4, 5], and the HR-competent breast cancer cell lines SKBR3 and SUM44 were employed as positive controls. Detection, image acquisition and analysis were performed as previously described [4, 5]. At least 100 nuclei in five separate high power fields were counted. A nucleus was considered RAD51 positive if it contained ≥ 5 discreet foci; results were presented as proportions of nuclei showing any Ki67 expression, as previously described [4, 5].

Immunoblotting

Whole cell lysates were generated from cells by incubation in HSE lysis buffer (20 mM HEPES, 200 mM NaCl, 1% Triton X-100, 1 mM DTT, 1 mM EDTA, 10% glycerol) with a protease inhibitor cocktail (Sigma Aldrich, St. Louis, MO, USA), and western blotting was performed as previously described [6], using anti-β-tubulin (ab6046, Abcam, Cambridge, UK), anti-PTEN (138G6, Cell Signaling Technology, Danvers, MA, USA), anti-BRCA2 (Ab-1 [OP95], Calbiochem/Merck, Nottingham, UK) and anti-BRCA1 (C-20, Santa Cruz Biotechnology) antibodies diluted in 5% milk/TBST. Incubation with a HRP-conjugated secondary antibody was then performed, and protein detected by chemiluminescence (AceGlo, Peqlab, Erlangen, Germany).

Sanger sequencing

Sequencing of the full-length cDNA of PTEN was performed for all cell lines as previously described [1]. RNA extraction using Trizol (Invitrogen, UK) and cDNA synthesis were performed as previously described [5]. 50 ng cancer cell line-derived cDNA was amplified and sequencing reactions were carried out using the DNA Sequencing Kit BigDye Terminator v 1.1 Cycle Sequencing Ready Reaction Mix (Applied Biosystems, UK), as previously described [1]. Sequences were analysed with Mutation Surveyor software (Softgenetics, USA). Mutations were confirmed by a repeat PCR from a new cDNA from the cell line harbouring the mutation and by sequencing of forward and reverse strands.

Assessment of *PTEN* copy number in OCCC cells

To determine *PTEN* copy number in OCCC cells and if any OCCC cells harboured homozygous deletions of *PTEN,* fluorescence *in situ* hybridisation (FISH) was performed as previously described [4, 7]. The Vysis *PTEN/CEP10* dual colour FISH probe (Abbott Molecular, Des Plaines, IL, USA) was hybridised to slides as previously described [8]. 60 non-overlapping nuclei harbouring *CEP10* signals were counted and cell lines were defined as harbouring homozygous deletions if nuclei lacked *PTEN* signals.

Cisplatin sensitivity assays

Cisplatin sensitivity assays were carried out in 96 well plates, in triplicate, as previously described [4]. OCCC cells were seeded at a density to allow at least seven days growth (1000–2000 cells/well), using CAPAN1 as a HR DNA repair-deficient and cisplatin-sensitive control [9–11]. Cells were treated with DMSO (control) or cisplatin (serial dilutions; 10^{-7} M to 10^{-4} M) (Sigma Aldrich) on days one and four following seeding. CellTiter Glo Luminescent Cell Viability Assay (Promega, UK) was used according to manufacturer's instructions to determine the survival fraction of cisplatin vs DMSO-treated cells.

BMN-673 sensitivity assays

BMN-673 sensitivity was determined using colony formation assays (CFAs) in six well plates in triplicates, as previously described [4]. Briefly, cells were seeded at a density to allow at least 10 days growth in six well plates (500–1000 cells/well). Media containing BMN-673 at concentrations ranging from 10^{-7} M to 10^{-11} M or DMSO alone were replaced every 48 hrs. After 14 days, cells were fixed in 1% TCA for one hour at 4ºC, stained with sulphorhodamine B, and absorbance measured to quantify the cell number. Background absorbance was deducted from each reading and values normalised to those obtained from DMSO-treated cells as previously described [4, 5].

Patient samples and tumour characteristics

Formalin-fixed paraffin-embedded (FFPE) samples from 50 consecutive primary OCCCs were retrieved from the pathology files of The Royal Marsden Hospital, London, The Edinburgh Royal Infirmary, The Royal Hospital Group, Belfast and The Hammersmith Hospital, London, as previously described [1]. Patient demographics and tumour characteristics are summarised in Supplementary Table S3, in line with REMARK guidelines (Supplementary Table S1). Samples were anonymised prior to analysis and the study approved by local ethical committees of the authors' institutions. All cases were reviewed and diagnosis of clear cell carcinoma confirmed as previously described[1]. Tissue microarrays (TMAs) were constructed from paraffin blocks with triplicate 0.6 mm tumour cores, containing 50 samples and normal tissue controls, as previously described [7]. Surgical and adjuvant therapy are described in Supplementary Table S3.

Immunohistochemistry (IHC)

Representative 3 μm-thick sections of the TMA described above were subjected to immunohistochemistry (IHC) using an antibody against PTEN (6H2.1 Dilution 1:100, Antigen retrieval, Dako target retrieval solution, pH9, 20 min 97°C DAKO, Glostrup, Denmark). The protocol for PTEN immunohistochemical analysis was previously validated [4]. Immunohistochemical analysis was interpreted by at least two pathologists (AC-F, AG and/ or JRF) using a semi-quantitative scoring system. Nuclear and cytoplasmic PTEN expression was scored using the Quick Score method [12], with cases defined as PTEN negative if nuclear expression was absent (i.e. Quick Score = 0). Cases with discordant scores between replicate cores, or those with missing cores from the TMA were assessed on full sections by the three pathologists on a multi-headed microscope and a consensus score for each was rendered.

Statistical analysis

All statistical analyses were carried out in Prism 6.05 (Graphpad Software Inc., California, USA). Drug sensitivity curves were constructed as previously described [13] using non-linear regression (curve fit) analysis, and SF_{50} (i.e. the drug concentration at which 50% of the cells survive compared to the vehicle-treated control cells) interpolated from these curves. Using the SF_{50} for each cell line as a surrogate, the relative sensitivities of HR competent and HR defective cell lines to cisplatin or BMN-673 were compared using a Mann-Whitney *U* test, as previously described [4, 5]. The proportions of RAD51-positive nuclei were compared between HR competent and HR defective cell lines using an unpaired, two-tailed Mann-Whitney *U* test. A two-tailed p value < 0.05 was considered significant.

REFERENCES

1. Tan DS, Lambros MB, Rayter S, Natrajan R, Vatcheva R, Gao Q, Marchio C, Geyer FC, Savage K, Parry S, Fenwick K, Tamber N, et al. PPM1D is a potential therapeutic target in ovarian clear cell carcinomas. Clin Cancer Res. 2009; 15:2269–80.
2. Nims RW, Sykes G, Cottrill K, Ikonomi P, Elmore E. Short tandem repeat profiling: part of an overall strategy for reducing the frequency of cell misidentification. *In Vitro* Cell Dev Biol Anim. 2010; 46:811–9.
3. Barallon R, Bauer SR, Butler J, Capes-Davis A, Dirks WG, Elmore E, Furtado M, Kline MC, Kohara A, Los GV, MacLeod RA, Masters JR, et al. Recommendation of short tandem repeat profiling for authenticating human cell lines, stem cells, and tissues. *In Vitro* Cell Dev Biol Anim. 2010; 46:727–32.
4. Dedes KJ, Wetterskog D, Mendes-Pereira AM, Natrajan R, Lambros MB, Geyer FC, Vatcheva R, Savage K, Mackay A, Lord CJ, Ashworth A, Reis-Filho JS. PTEN deficiency in endometrioid endometrial adenocarcinomas predicts sensitivity to PARP inhibitors. Science translational medicine. 2010; 2: 53ra75.
5. Wilkerson PM, Dedes KJ, Wetterskog D, Mackay A, Lambros MB, Mansour M, Frankum J, Lord CJ, Natrajan R,

Ashworth A, Reis-Filho JS. Functional characterisation of EMSY gene amplification in human cancer. J Pathol. 2011; 225:29–42.

6. Natrajan R, Lambros MB, Rodriguez-Pinilla SM, Moreno-Bueno G, Tan DS, Marchio C, Vatcheva R, Rayter S, Mahler-Araujo B, Fulford LG, Hungermann D, Mackay A, et al. Tiling path genomic profiling of grade 3 invasive ductal breast cancers. Clin Cancer Res. 2009; 15:2711–22.
7. Tan DS, Iravani M, McCluggage WG, Lambros MB, Milanezi F, Mackay A, Gourley C, Geyer FC, Vatcheva R, Millar J, Thomas K, Natrajan R, et al. Genomic analysis reveals the molecular heterogeneity of ovarian clear cell carcinomas. Clin Cancer Res. 2011; 17:1521–34.
8. Lambros MB, Simpson PT, Jones C, Natrajan R, Westbury C, Steele D, Savage K, Mackay A, Schmitt FC, Ashworth A, Reis-Filho JS. Unlocking pathology archives for molecular genetic studies: a reliable method to generate probes for chromogenic and fluorescent *in situ* hybridization. Lab Invest. 2006; 86:398–408.
9. Edwards SL, Brough R, Lord CJ, Natrajan R, Vatcheva R, Levine DA, Boyd J, Reis-Filho JS, Ashworth A. Resistance to therapy caused by intragenic deletion in BRCA2. Nature. 2008; 451:1111–5.
10. Bhattacharyya A, Ear US, Koller BH, Weichselbaum RR, Bishop DK. The breast cancer susceptibility gene BRCA1 is required for subnuclear assembly of Rad51 and survival following treatment with the DNA cross-linking agent cisplatin. J Biol Chem. 2000; 275:23899–903.
11. Hastak K, Alli E, Ford JM. Synergistic chemosensitivity of triple-negative breast cancer cell lines to PARP inhibition, gemcitabine and cisplatin. Cancer Res. 2010; 70:7970–80.
12. Harvey JM, Clark GM, Osborne CK, Allred DC. Estrogen receptor status by immunohistochemistry is superior to the ligand-binding assay for predicting response to adjuvant endocrine therapy in breast cancer. J Clin Oncol. 1999; 17:1474–81.
13. Farmer H, McCabe N, Lord CJ, Tutt AN, Johnson DA, Richardson TB, Santarosa M, Dillon KJ, Hickson I, Knights C, Martin NM, Jackson SP, et al. Targeting the DNA repair defect in BRCA mutant cells as a therapeutic strategy. Nature. 2005; 434:917–21.

Supplementary Table S1: Clinicopathological information of primary OCCC

**Age**	**OCCC (N = 50)**
Mean (years)	58.1
Range (years)	34–82
**Predominant pattern**	
Glandular	8 (16%)
Papillary	15 (30%)
Solid	12 (24%)
Tubulocystic	15 (30%)
**Mitotic count (per 10 hpf)**	
Mean	5.3
Median	2
Range	1–40
**Necrosis**	
Yes	35 (70%)
No	15 (30%)
**Inflammation**	
Yes	11 (22%)
No	39 (78%)
**Adenofibroma-like**	
Yes	10 (20%)
No	40 (80%)
**Debulking surgery**	
Optimal	41 (82%)
Suboptimal	7 (14%)
Unknown	2 (4%)
**Chemotherapy**	
None	10 (20%)
Platinum salt alone	14 (28%)
Platinum salt + Taxane	26 (52%)
**Follow up**	
Median (months)	23.95
Range (months)	1.2–159.6
**PTEN expression (n = 49)**	
Absent	4 (8.2%)
Present	45 (91.8%)

Supplementary Table S2: Compliance with REMARK guidelines

REporting recommendations for tumour MARKer prognostic studies (REMARK)	How addressed
**Introduction**<br>1 State the marker examined, the study objectives and any prespecified hypotheses	Defined in introduction
**Material and methods**	
Patients	
2 Describe the characteristic (e.g. disease stage or comorbidities) of the study patients, including their source and inclusion and exclusion criteria	Referenced, and defined in Materials and Methods and Supplementary Table S1
3 Describe the treatments received and how chosen (e.g. randomized or rule-based)	
Specimen characteristics	
4 Describe the type of biological material used (including control samples) and methods of preservation and storage	Referenced, and defined in Materials and Methods and Supplementary Table S1
Assay methods	
5 Specify the assay method used and provide (or reference) a detailed protocol, including specific reagents or kits used, quality control procedures, reproducibility assessments, quantification methods, and scoring and reporting protocols. Specify whether and how assays were performed blinded to the study endpoint	Defined in Materials and Methods
Study design	
6 State the method of case selection, including whether prospective or retrospective and whether stratification or matching (e.g. by stage of disease or age) was used. Specify the time period from which cases were taken, the end of the follow-up period, and the median follow-up time.	Referenced, and defined in Materials and Methods and Supplementary Table S1
7 Precisely define all clinical endpoints examined	Not applicable
8 List all candidate variables initially examined or considered for inclusion in models	
9 Give rationale for sample size; if the study was designed to detect a specified effect size, give the target power and effect size.	
Statistical analysis methods	
10 Specify all statistical methods, including details of any variable selection procedures and other model-building issues, how model assumptions were verified, and how missing data were handled	Defined in Materials and Methods
11 Clarify how marker values were handled in the analyses; if relevant, describe methods used for cutpoint determination	Defined in Materials and Methods
**Results**	
Data	
12 Describe the flow of patients through the study, including the number of patients included in each stage of the analysis (a diagram may be helpful) and reasons for dropout. Specifically, both overall and for each subgroup extensively examined report the numbers of patients and the number of events	Not applicable
13 Report distributions of basic demographic characteristics (at least age and sex), standard (disease-specific) prognostic variables, and tumour marker, including numbers of missing values	Referenced, and defined in Materials and Methods and Supplementary Table S1
Analysis and presentation	
14 Show the relation of the marker to standard prognostic variables	Shown in Results
15 Present univariate analyses showing the relation between the marker and the outcome, with the estimated effect (e.g. hazard ratio and survival probability). Preferably provide similar analyses for all other variable being analyzed. For the effect of a tumour marker on a time-to-event outcome, a Kaplan-Meier plot is recommended	Not applicable
16 For key multivariable analyses, report estimated effects (e.g. hazard ratio) with confidence intervals for the marker and, at least for the final model, all other variables in the model	
17 Among reported results, provide estimated effects with confidence intervals from and analysis in which the marker and standard prognostic variables are included, regardless of their statistical significance	
18 If done, report results of further investigations, such as checking assumptions, sensitivity analyses, and internal validation	Defined in Materials and Methods
**Discussion**	
19 Interpret the results in the context of the prespecified hypotheses and other relevant studies; include a discussion of limitations of the study	Reported in Discussion
20 Discuss implications for future research and clinical value	Reported in conclusion

Adapted from McShane et al (Nature Clinical Practice Oncology 2005; 2: 416–422).

Supplementary Table S3: *BRCA1/2*, *PTEN* and *TP53* mutations detected in present panel of OCCC cell lines

Cell Line	BRCA1*	BRCA2[3]	PTEN1	TP53[1]
ES-2[7)]	wt	wt	wt	241 S > F (722 C > T)
HCH-1	n/a	n/a	wt[4]	wt[4]
KK	wt	wt	wt	273 R > H (818 G > A)
KOC-7c	c.395A > C[3]<br>c.39T > A[3]	n/a	89707652C > CT:233R>R/X[3] (c.968het_delA)*<br>89710792het_delA[3] (c.697C > CT)*	n/a
OVAS	c.5343C > G	n/a	n/a (wt)	n/a
OVISE[7)]	wt	wt	wt	wt
OVMANA[7)]	wt	c.2275 C > G	wt	wt
OVSAYO	n/a	n/a	n/a (wt*)	wt[6]
OVTOKO[7)]	wt	wt	wt	wt
RMG-1[7)]	wt	wt	wt	wt
SMOV-2	n/a	n/a	n/a (wt*)	wt[5]
TOV-21G	wt	wt	89682921 delG (425 delG)[1,3,*]<br>89707755 delA (795delA)[1,3,*]	wt

Source:

*)own data

1)Shih IeM, Am J Pathol. 2009.
2)R. Miller, Mol Cancer Ther; 15 (7) July 2016.
3)Ihnen M, Mol Cancer Ther; 12 (6) June 2013.
4)Yamada et al. Journal of Ovarian Research (2016) 9:32.
5)Yonamine K. et al., Hum Cell, 1999.
6)Creighton CJ et al., Cancer Res, 2010.
7)S. Domcke, Nature com. 2013.

KOC7C: data n/a (Pubmed/Medline, CCLE, canSAR).
